# An algorithm for power transmission line fault detection based on improved YOLOv4 model

**DOI:** 10.1038/s41598-024-55768-1

**Published:** 2024-02-29

**Authors:** Su Yan, Lisha Gao, Wendi Wang, Gang Cao, Shuo Han, Shufan Wang

**Affiliations:** 1 Nanjing Suyi Industry Co., Ltd, Nanjing, 210000 Jiangsu Province China; 2 Nanjing Power Supply Branch, State Grid Corporation of Jiangsu Province, Nanjing, 210009 Jiangsu Province China

**Keywords:** Fault detection, Grouped convolution, Network optimization, YOLOv4, Computer science, Power distribution

## Abstract

In response to the escalating demand for real-time and accurate fault detection in power transmission lines, this paper undertook an optimization of the existing YOLOv4 network. This involved the substitution of the main feature extraction network within the original YOLOv4 model with a lighter EfficientNet network. Additionally, the inclusion of Grouped Convolution modules in the feature pyramid structure replaced conventional convolution operations. The resulting model not only reduced model parameters but also effectively ensured detection accuracy. Moreover, in enhancing the model's reliability, data augmentation techniques were employed to bolster the robustness of the power transmission line fault detection algorithm. This optimization further utilized the DIoU loss function to stabilize target box regression. Comparative experiments demonstrated the improved YOLOv4 model's superior performance in terms of loss function optimization while significantly enhancing detection speed under equivalent configurations. The parameter capacity was reduced by 81%, totaling merely 43.65 million, while the frame rate surged by 85% to achieve 24 frames per second. These experimental findings validate the effectiveness of the algorithm.

## Introduction

Power transmission lines are a crucial component of the electrical power system, extensively used in power transmission and distribution. Various faults are inevitable during the operation of transmission line, and these faults can significantly affect the stability and safety of the power system. Therefore, accurate and rapid automatic detection of power transmission lines is essential. Efficient fault detection technology enables the timely identification and resolution of issues in transmission lines, ensuring the continuity and reliability of power supply.

The advancement of machine learning has led to its application in identifying the fault of power transmission line, which can be divided into two main categories. The first category includes those that consider the generated time-series voltage and current signal waveforms from generators to be fed to the traditional machine learning algorithms. Aker et al.^1^ utilized the Daubechies mother wavelet from the Wavelet Daubechies (db4) series to break down the three-phase fault current waveforms into multiple levels. This process helped in extracting features like standard deviation and energy values. In 2019, Chaitanya et al.^2^ introduced a method involving a decision tree supported by traveling wave-based techniques for multi-terminal transmission lines. Fonseca et al.^3^ demonstrated the application of the random forest with an initial preprocessing step involving a notch filter to accurately identify different fault types in transmission lines. The same year, Ghashghaei and Akhbari^4^ offered a comprehensive ML-based framework for diagnosing faults in transmission lines, employing various machine learning algorithms like SVM and K-NN. Moreover, the second category includes those with focus on the image-based datasets taken from outdoor transmission line. In recent years, with the increase in the availability of image data, several studies^5–7^ have focused on the detection, classification, and pinpointing of transmission line faults, employing Convolutional Neural Networks (CNNs) to enhance accuracy.

However, these models, due to their reliance on manually designed feature extractors, suffered from slow training and reasoning speeds, low accuracy, and poor cross-domain performance^8^. This makes them less suitable for application in transmission line fault detection, where efficient and real-time accuracy plays a crucial role. The introduction of Convolutional Neural Networks (CNNs) has significantly enhanced the performance of object detection algorithms. For instance, Fast R-CNN^9^ ingeniously integrates feature extraction, classification, and localization operations, substantially improving the effectiveness of object training and prediction. With the advent of Faster R-CNN, the concept of candidate regions was introduced, further enhancing the precision and speed of object detection^10^, and offering robust support for practical applications. Additionally, the YOLO network innovatively employs a regression method^11^, enabling simultaneous object classification and localization, significantly boosting detection efficiency. Although its accuracy is slightly lower than that of Faster R-CNN (e.g., Redmon and Farhadi^11^ used the VOC 2007 public dataset to compare the classification accuracy of Faster R-CNN and YOLO, and found that Faster R-CNN had a 6.1% higher accuracy than YOLO), YOLO holds unique advantages in real-time detection. It is capable of swiftly capturing targets in fast-moving scenarios, providing a powerful solution for applications requiring immediate feedback and high-speed object detection.

We chose YOLOv4 for its proven reliability and stable performance, evidenced by its adoption in various detection tasks. Despite the existence of newer versions like YOLOv8, YOLOv4 remains preferable for power transmission line fault detection due to its accuracy and consistency. The YOLOv4 algorithm has gained widespread application in current research, notably in crop detection^12^, orchard pests detection^13^ and vehicle recognition^14^ using its backbone network. Other study modified the CSPDarkNet53 backbone network of YOLOv4 to an inverted residual structure for automated aluminum surface defect detection^8^. However, the application of this technology in power transmission line fault detection is still restricted. In this particular context, the limitations of hardware platforms pose challenges to the seamless deployment of high-capacity, high-speed detection model. To address this issue, our study utilizes the lightweight EfficientNet network to replace the main feature extraction network of the original model and incorporates grouped convolution modules in the feature pyramid structure. This enhances the efficiency and accuracy of fault detection in power transmission lines. The improved method combines the speed and high precision advantages of the YOLOv4 model, reduces memory usage, and simultaneously enhances the real-time capability of fault detection in power transmission lines.

Compared to existing research, our study offers the following contributions. Firstly, to our knowledge, this is the first paper to validate the application of the YOLOv4 algorithm in the field of power transmission line fault detection using large-scale image data. Specifically, we utilized over a million unique image data collected from the State Grid Corporation of China as training samples, thereby expanding the potential applications of the YOLOv4 algorithm. Secondly, this research replaces the primary feature extraction network in the original object detection model with the lightweight EfficientNet network. Additionally, we have integrated grouped convolution modules within the feature pyramid structure, significantly enhancing the efficiency and accuracy of the YOLOv4 algorithm in diagnosing faults in power transmission lines.

## YOLOv4 model improvements

### EfficientNet-based network replacement

An essential function of the EfficientNet network is feature extraction^15^, which can preliminarily extract features from image patches through the main network in YOLOv4. Typically, neural networks perform feature extraction using convolution operations, gradually reducing the width and height of feature layers while increasing the number of channels. Notably, the EfficientNet network exhibits an impressive characteristic by resembling the feature layer's width and height of the CSPDarknet53 network. This similarity positions the EfficientNet as an ideal replacement for the CSPDarknet53 network. Leveraging the similarity in the width and height of feature layers between EfficientNet and CSPDarknet53, this paper introduces the EfficientNet into YOLOv4 to substitute the original CSPDarknet53 network.

The primary characteristic of the EfficientNet network lies in its use of compound coefficients to balance the network's complexity and performance. By employing these compound coefficients, the network adjusts its width, depth, and resolution to achieve efficient and high-performance feature extraction. The fundamental structure of EfficientNet involves depth-wise separable convolutions in the width dimension and expandable convolutions in the depth dimension, accompanied by an attention mechanism to enhance feature representation capability. In the EfficientNet, the backbone amplifies the channel numbers of the input feature layer using 1 × 1 convolutions and employs depth-wise separable convolutions in the width direction for feature extraction. Depth-wise separable convolutions segregate traditional convolutions into depth convolutions and pointwise convolutions, thereby reducing computational load and the number of parameters. Leveraging the EfficientNet network allows for capitalizing on the advantages of its compound coefficients and attention mechanism, enabling efficient feature extraction and robust feature representation capability. This will contribute to enhancing the detection accuracy of the YOLOv4 model while maintaining fewer model parameters, thereby improving the model's real-time performance and applicability.

### Grouped convolution module replaces traditional convolution

**Figure 1:**
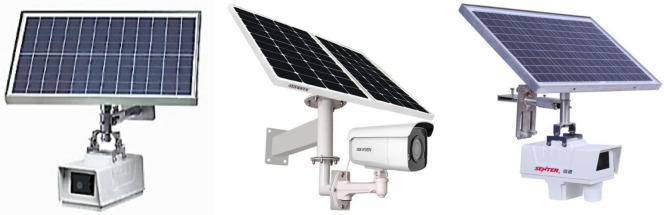
Visual intelligent terminal for the detection of transmission line faults.

China primarily utilizes the visual intelligent terminals shown in the Fig. 1 for the detection of transmission line faults. These devices predominantly rely on solar energy for power, imposing stringent constraints on energy consumption and computational capacity. In practical settings, the computational power of these terminals is approximately 15 TOPS, subject to variations in real-world scenarios.

This paper proposes the use of Grouped Convolution (GConv) modules to replace traditional depth-wise separable convolutions, aiming to achieve efficient feature extraction and significantly reduce the model's parameter count. In standard convolution within convolutional neural networks (CNNs), filters extract features by performing element-wise multiplications on multi-channel input data. In contrast, Grouped Convolution alters this process by dividing input channels into multiple groups. For example, 32 channels can be split into 4 groups, each containing 8 channels, where each filter interacts only with the channels within its designated group. This approach reduces the number of parameters in the convolutional layers, enhancing the network's computational efficiency and allowing for an expansion of the network structure without increasing computational overhead. Grouped Convolution enhances feature diversity by processing features in parallel across different groups. Although this might decrease the interaction between features across different groups, it opens new pathways for improving overall network performance and learning capabilities.

Grouped Convolution represents a lightweight convolution operation that offers greater flexibility in controlling computational load and parameter count, making it well-suited for efficient feature extraction. In PANet, after merging feature layers through upsampling and downsampling fusion, multiple applications of Grouped Convolution are employed to fuse and extract features, producing effective feature layers. Unlike depth-wise separable convolutions, Grouped Convolution divides input channels into groups, conducting independent convolution operations within each group, then concatenates the outputs from these groups. This method substantially reduces model parameters and computational load, focusing PANet's parameters primarily on Grouped Convolution operations. By substituting Grouped Convolution for traditional 3 × 3 depth-wise separable convolutions, this strategy successfully decreases the parameter count in PANet. This optimization not only maintains effective feature extraction but also enhances the model's operational speed, catering to resource-constrained environments, better meeting the real-time and accuracy requirements of power transmission line fault detection.

### Improvement of position regression loss function

When evaluating object detection algorithms, Intersection over Union (IOU) is commonly used to measure the degree of overlap between predicted and actual bounding boxes. However, the newly introduced Distance Intersection over Union (DIoU) builds upon IOU by considering the distance between the centers of predicted and actual bounding boxes. This measure is integrated into the regression component of the loss function to further optimize the model's performance^16^. The DIoU loss function not only computes the overlap between predicted and actual bounding boxes but also accounts for the distance between their centers. By incorporating DIoU into the loss function, the model not only focuses on the alignment of bounding box positions during optimization but also emphasizes the optimization of center point distances, thereby further enhancing detection accuracy. The DIoU formula is as follows:

1 $$\begin{array}{c}DIoU=IoU-\frac{{\rho }^{2}\left(b,{b}^{gt}\right)}{{c}^{2}}=IoU-\frac{{d}^{2}}{{c}^{2}}\end{array}$$

In the equation: ${\rho }^{2}\left(b,{b}^{gt}\right)$ represents the distance between the centers of the two boxes, while $c$ denotes the diagonal distance of the smallest enclosing region that encompasses both boxes.

## Experimental results and analysis

### Experimental environment establishment

**Figure 2:**
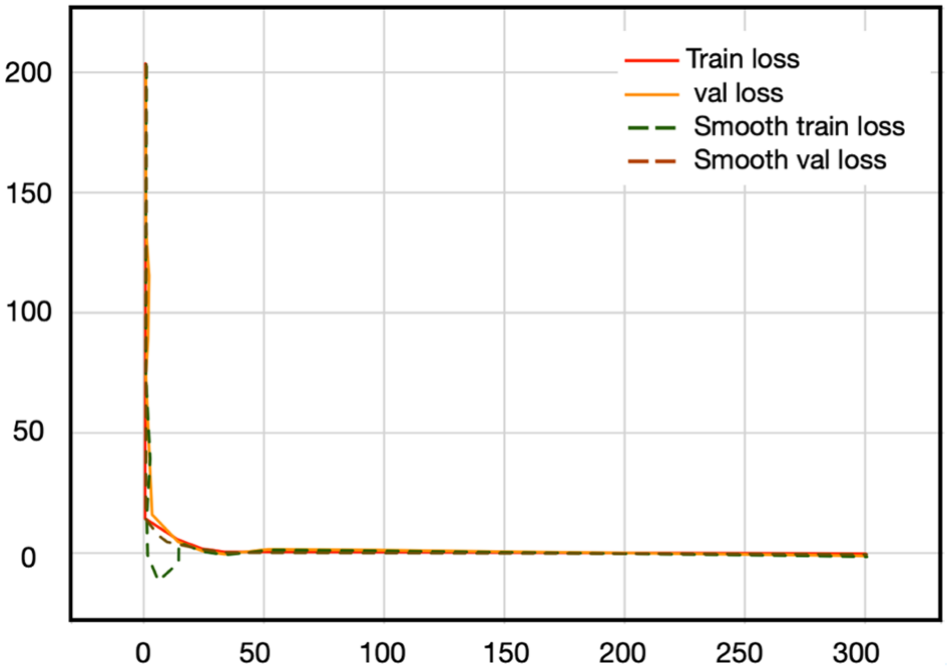
Graph of loss function.

This paper selected PyCharm 2022.2.2 as the editing tool and constructed the PyTorch 2.2 deep learning framework as the experimental environment, dividing training and testing samples in a 9:1 ratio. To enhance the robustness of the detection algorithm, Mosaic data augmentation was employed during the training process, and the cosine annealing decay strategy was used to adjust the learning rate. This strategy emphasizes the simulated cosine function descent to manage the decrease in learning rates and linear ascent to handle learning rate increments. Additionally, Label Smoothing techniques were applied to prevent overfitting phenomena. Figure 2 illustrates the loss function curve of the model after a single training iteration, with "train loss" representing the loss function of the training dataset, and "val loss" indicating the loss function of the validation dataset. Similarly, the smoothed loss functions of the training and validation datasets were labeled as "smooth train loss" and "smooth val loss." At the initial stages of training, there was significant deviation and negative values in the loss function due to the lack of data normalization and the absence of smoothed labels in the training set. These issues were resolved as subsequent training iterations progressed. As depicted in the figure, after 100 epochs, the model's loss function values converged, with 150 chosen as the final epoch number to ensure the stability of the training state. This process demonstrates that some fluctuations may occur during the initial training phase, but over time, the model gradually learns to better adapt to the input data, eventually reaching a satisfactory performance level. The input shape dimensions were 416 × 416. The frozen training method not only ensures stable weights in the model's initial training phase but also accelerates the training speed. When the model is in a frozen state, it aids in saving memory resources and maintains the integrity of the feature extraction network. However, when the model is in an unfrozen state, noticeable fluctuations in the parameters of the feature extraction network and a substantial increase in called memory resources occur. Therefore, this paper adopted the frozen training method to achieve optimization effects. Considering the current hardware platform conditions, this paper set the batch size during the unfrozen stage to 4 and during the frozen stage to 8. To expedite data retrieval, a multi-threaded data reading approach was utilized.

### Result analysis

#### Comparison of parameters and FPS

This paper conducted a series of comparative experiments to evaluate the advantages of the proposed algorithm model in terms of simplicity and speed compared to commonly used object detection models, including YOLOv3, YOLOv4, and MobilenetV3-YOLOv4. The experimental results in Table 1 demonstrate that in terms of parameter count and capacity, YOLOv3 and YOLOv4 exhibited only a minor increase compared to previous versions, with counts of 6.08 × 10^7^ and 6.38 × 10^7^ and capacities of 227.88 M and 238.62 M, respectively. However, the algorithm proposed in this paper significantly reduced the parameter count and capacity by 80% and 81%, resulting in counts of 1.21 × 10^7^ and a capacity of 43.65 M. Moreover, the algorithm of this paper demonstrated excellent performance in frame rate, achieving 24 frames/s, a 60% and 85% improvement compared to the frame rates of 15 frames/s and 13 frames/s when using the YOLOv3 and YOLOv4 algorithms, respectively. It is noteworthy that MobilenetV3-YOLOv4 had a parameter count of 1.29 × 10^7^, a capacity of 50.13 M, and a frame rate of 22 frames/s, while the proposed algorithm in this paper had a parameter count of 1.31 × 10^7^, a capacity of 43.65 M, and a frame rate of 24 frames/s, indicating higher performance and efficiency. Although this research's algorithm, like the MobilenetV3-YOLOv4 algorithm, replaced the core feature extraction network, this paper further enhanced the model's efficiency using attention techniques, resulting in a 6.2% reduction in parameter count and a 9.1% increase in frame rate. This effectively validates the advantages of this paper's algorithm in model capacity and detection speed. Considering the strict limitations on energy consumption and computational capacity of transmission line fault detection devices, our algorithm holds significant practical importance.

**Table 1:** Parameter and FPS comparison.

Algorithm	Parameter quantity	Parameter capacity/MB	FPS/(frame/S)
YOLOv3	6.08 × 10^7^	227.88	15
YOLOv4	6.38 × 10^7^	238.62	13
MobilenetV1-YOLOv4	1.29 × 10^7^	50.13	22
Our algorithm	1.21 × 10^7^	43.65	24

#### Accuracy comparison

**Figure 3:**
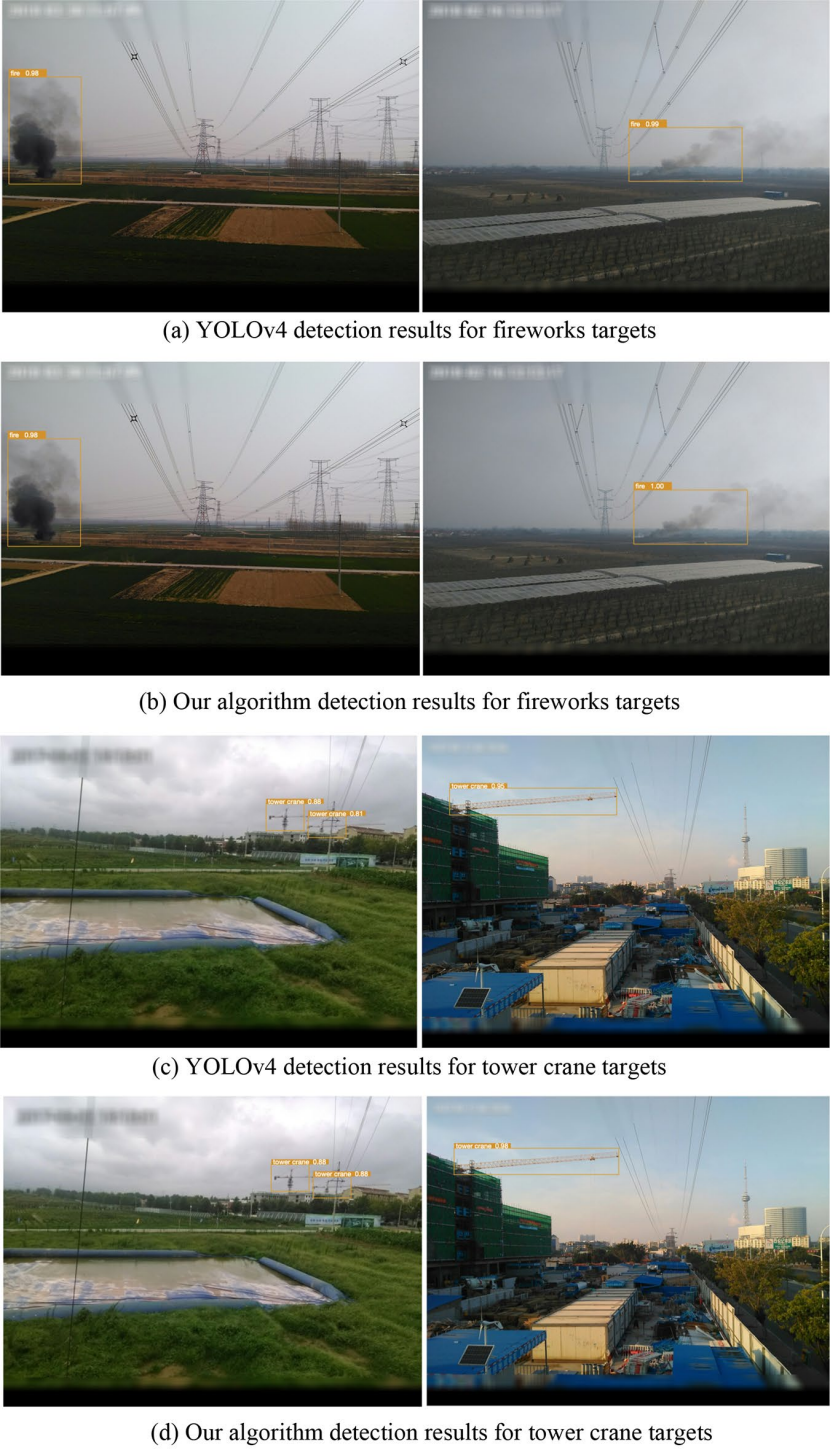

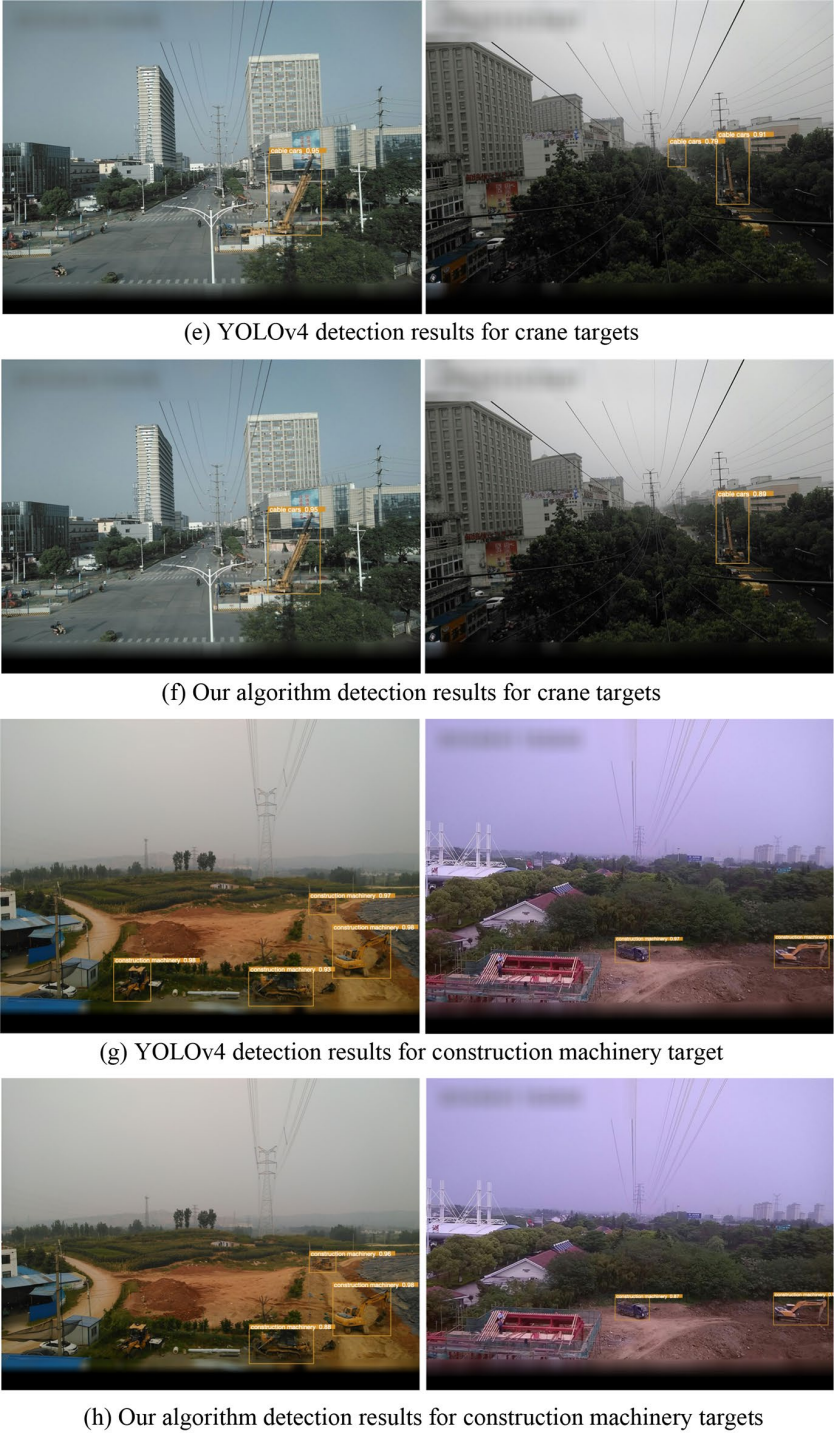
Detection results under different scenarios (YOLOv4 vs. our algorithm).

This paper conducted comparative experiments involving various categories of objects such as cranes, tower cranes, fireworks, and construction machinery, to assess different recognition types. This approach aimed to avoid the instability of results in scenarios involving the identification of single-class objects. Some detection results are illustrated in the following Fig. 3:

It's evident that both algorithms exhibit generally lower accuracy in recognizing cranes and construction machinery. This could be attributed to the presence of numerous vehicles on the road with similar appearances or body structures, posing challenges for identification. On the other hand, fireworks and tower cranes, having distinct features and being less prone to interference from other similar objects, show minimal differences in accuracy between the two algorithms and demonstrate good performance. Overall, while this algorithm enhances detection speed, it does sacrifice a certain degree of accuracy, which is deemed acceptable. Further detailed comparisons of object detection accuracy across different algorithms are outlined in Table 2.

**Table 2:** Comparison of transmission line fault detection accuracy under different algorithms.

Algorithm	Fireworks			Tower crane			Crane			Construction machinery			mAP%
	AP%	R%	P%	AP%	R%	P%	AP%	R%	P%	AP%	R%	P%	
YOLOv4	96.3	96.0	96.2	94.5	92.1	96.1	88.6	85.2	90.7	87.9	83.1	90.3	91.8
Our algorithm	96.1	95.3	96.2	92.7	90.3	93.7	85.2	82.9	87.6	85.3	79.6	89.2	89.8

In the detection of fireworks, the improved algorithm exhibited a decrease in recall by 0.7%. For tower crane detection, the improved algorithm showed a decrease in precision by 2.4% and a decrease in recall by 1.8%. In the case of crane detection, the improved algorithm demonstrated a reduction in precision by 3.1% and a decrease in recall by 2.3%. For construction machinery detection, the improved algorithm displayed a decrease in precision by 1.1% and a decrease in recall by 3.5%. From these results, it can be concluded that using the grouped convolution module reduces the parameter count but is also accompanied by a slight decline in model accuracy, especially in complex target scenarios. This is because target features in complex scenes are more intricate, requiring more precise parameters for feature extraction. In contrast, for simple target images, the impact of reducing parameter count on accuracy is relatively minor.

The improved performance of the proposed algorithm is attributed to several key advancements. Firstly, the integration of a lightweight EfficientNet network for feature extraction enhances the balance between accuracy and computational efficiency. Secondly, the use of Grouped Convolution in the feature pyramid structure significantly reduces the model's parameter count, making it more efficient. Thirdly, the incorporation of the DIoU loss function improves bounding box regression accuracy. Additionally, the application of data augmentation techniques bolsters the model's robustness and real-world applicability.

Our algorithm, significantly lighter than YOLOv4 in parameters (19% and 18%), has a slight 2% lower mAP but is more feasible for real-world deployment due to hardware constraints, as shown in Fig. 1, thus offering substantial practical value. The model achieves a notable improvement in detection speed with minimal accuracy loss, making it particularly suitable for real-time applications like fault detection in power transmission lines, where both speed and precision are critical.

However, our algorithm demonstrates a trade-off between increased detection speed and a slight reduction in accuracy. This trade-off is crucial in real-time applications like infrastructure monitoring, where quick detection is prioritized to prevent serious issues. However, the impact of reduced accuracy, potentially leading to false negatives or positives, is a critical consideration, as it can affect the reliability of the system. False negatives could result in undetected faults, while false positives might cause unnecessary actions. Balancing speed with accuracy is essential, necessitating ongoing evaluation and refinement to ensure the model's practical effectiveness in such sensitive applications.

## Conclusion

To meet the growing demands for enhanced performance, this paper introduces an improved YOLOv4 network model aimed at enhancing the real-time detection and precision of faults in power transmission lines. Within this enhanced model, a lightweight EfficientNet network replaces the original model's primary feature extraction network. Additionally, the model employs the Grouped Convolution module to construct the feature pyramid structure, replacing traditional convolutions. This approach ensures both detection accuracy and effectively reduces the model's parameter count. Furthermore, to enhance the stability of bounding box regression, this study incorporates the DIoU loss function and data augmentation techniques, thus bolstering the robustness of the object detection algorithm.

The comparative experimental results across various recognition scenarios reveal that, with equivalent hardware configurations, the improved model in this paper significantly enhances detection speed while experiencing only a marginal decrease in model accuracy. These empirical findings robustly validate the effectiveness of the proposed algorithm, providing strong support for its practical application in the detection of faults in power transmission lines.

Finally, it should be noted that integrate the DIoU loss function into the YOLOv4 model for object detection introduces additional computational complexity, particularly affecting bounding box regression accuracy. To manage this in power transmission line fault detection, especially in resource-limited settings, several strategies can be employed. Efficient coding and implementation of DIoU can reduce its computational burden, and optimizing the algorithm to leverage hardware accelerations, like GPUs, is beneficial. Employing a lightweight architecture such as EfficientNet in the feature extraction layer helps offset DIoU's extra load. In highly constrained environments, selectively using DIoU where precision is critical, and opting for simpler loss functions elsewhere, is recommended. Hardware optimization, model quantization, and pruning are also suggested to manage increased computational demands and reduce model size. Furthermore, edge computing, processing data locally on devices, and asynchronous processing, where DIoU calculations are deferred to less critical times, can also effectively balance computational efficiency and detection performance.

## Data Availability

The data that support the findings of this study are available from Nanjing Suyi Industrial Co., Ltd and State Grid Jiangsu Electric Power Co., Ltd., but restrictions apply to the availability of these data, which were used under license for the current study, and so are not publicly available. Data are however available from the authors upon reasonable request and with permission of Nanjing Suyi Industrial Co., Ltd. and State Grid Jiangsu Electric Power Co., Ltd.
